# Supplementary material for: Comparative ACE2 variation and primate COVID-19 risk
Source: Commun Biol. 2020 Oct 27;3:641. doi: 10.1038/s42003-020-01370-w (PMC7591510; doi:10.1038/s42003-020-01370-w)
Supplement: Supplementary file 1 — Supplementary Information [file 42003_2020_1370_MOESM1_ESM.pdf]

Supplementary Tables and Figures

Comparative ACE2 variation and primate COVID-19 risk

Melin AD, Janiak MC, Marrone F, Arora PS & Higham JP

Supplementary Table S1. Results of alanine scanning mutagenesis experiments predicting critical binding sites between ACE2 and Sars-CoV-2 receptor binding domain. Residues whose mutation to alanine decrease the binding energy by $\Delta\Delta G_{bind}$ ≥1.0 kcal/mol are considered to be significant for binding (in bold).

Residue site	ΔΔG (kcal/mol)[a]
**41[+]**	**4.1**
**355**	**3.5**
**42[+]**	**2.3**
**83**	**2.1**
**357[+]**	**2**
**38**	**1.4**
**37**	**1.2**
**24[+]**	**1.1**
**353[+]**	**1.1**
27	0.7
34[+]	0.7
31	0.6
30[+]	0.6
35	0.5
79	0.5
45	0.5
28	0.3
82[+]	0.2
330	0.2
351	0.03

[a]The computational alanine mutagenesis analysis was performed with Rosetta Software and PDB file 6M0J.

[+]Denotes sites also implicated by Yan et al.[1]

Supplementary Table S2. Gene IDs and NCBI accession numbers of *ACE2* gene sequences included in the study.

**Species**	**Common Name**	**Gene ID**	**NCBI Accession Number**	**Notes**
Homo sapiens	human	59272	NM_001371415.1	
Pan troglodytes	chimpanzee	465511	XM_016942979.1	
Pan paniscus	bonobo	100970340	XM_008974180.1	
Gorilla gorilla	gorilla	101142534	XM_019019204.1	
Pongo abelii	Sumatran orangutan	100171441	NM_001131132.2	
Nomascus leucogenys	northern white-cheeked gibbon	100602708	XM_003261084.3	
Hylobates moloch	silvery gibbon	116811532	XM_032756617.1	
Rhinopithecus roxellana	golden snub-nosed monkey	104664530	XM_010366065.2	
Piliocolobus tephrosceles	Ugandan red colobus	111531712	XM_023199053.2	
Macaca mulatta	rhesus macaque	712790	NM_001135696.1	
Macaca nemestrina	pigtail macaque	105478157	XM_011735203.2	
Macaca fascicularis	long-tailed macaque	102130864	XM_005593037.2	
Cercocebus atys	sooty mangabey	105574684	XM_012035809.1	
Mandrillus leucophaeus	drill	105550583	XM_011995533.1	
Papio anubis	olive baboon	101008749	XM_021933040.1	
Theropithecus gelada	gelada	112615413	XM_025372062.1	
Chlorocebus sabaeus	vervet	103231639	XM_007991113.1	
Alouatta palliata	mantled howler monkey	N/A	N/A	unpublished draft genome, sequence in supplemental file
Aotus nancymaae	Ma’s night monkey	105705080	XM_012434682.2	
Cebus capucinus imitator	white-faced capuchin	108291904	XM_017512376.1	
Sapajus apella	tufted capuchin	116556688	XM_032285963.1	
Saimiri boliviensis	Bolivian squirrel monkey	101045190	XM_010336623.1	
Callithrix jacchus	common marmoset	100408882	XM_017968359.1	
Carlito syrichta	Philippine tarsier	103267011	XM_008064619.1	
Microcebus murinus	gray mouse lemur	105882317	XM_020285237.1	Exon 15 manually corrected, NCBI annotation incorrect
Propithecus coquereli	Coquerel's sifaka	105805773	XM_012638731.1	
Otolemur garnettii	Northern greater galago	100951881	XM_003791864.2	
Eulemur flavifrons	blue-eyed black lemur	N/A	LGHW01000591.1, scaffold 590	*ACE2* not annotated, identified via BLAST
Daubentonia	aye-aye	N/A	PVJZ01006595.1,	*ACE2* not annotated,

madagascariensis			scaffold 13170	identified via BLAST
Rhinolophus sinicus	Chinese rufous horseshoe bat	N/A	GQ999933.1	From [2]
Rhinolophus pusillus	least horseshoe bat	N/A	GQ999938.1	
Rhinolophus macrotis	big-eared horseshoe bat	N/A	GQ999932.1	
Rhinolophus pearsonii	Pearson's horseshoe bat	N/A	EF569964.1	
Rhinolophus ferrumequinum	greater horseshoe bat	N/A	GQ999931.1	
Myotis daubentonii	Daubenton's bat	N/A	GQ999937.1	
Hipposideros pratti		N/A	GQ999934.1	
Felis catus	domestic cat	554349	NM_001039456.1	
Canis lupus	domestic dog	480847	NM_001165260.1	
Sus scrofa	domestic pig	100144303	NM_001123070.1	
Mustela putorius	ferret	101673097	NM_001310190.1	
Manis javanica	Malayan pangolin	108390919	XM_017650257.1	

Supplementary Table S3. Full results of the codeml analyses of adaptive evolution across *ACE2* gene sequences.

codeml analysis	Foreground branch	Model	kappa	treeLength	number of parameters	omega	proportion	lnL	LRT	p	positively selected sites
branch-site model	platyrrhine	null	3.08706	3.33363	79	background: ω0 = 0.07549, ω1 = 1.00000, ω2a = 0.07549, ω2b = 1.00000; foreground: ω0 = 0.07549, ω1 = 1.00000, ω2a = 1.00000, ω2b = 1.00000	p0 = 0.63315, p1 = 0.35752, p2a = 0.00596, p2b = 0.00337	-15583.14271			
branch-site model	platyrrhine	alternative	3.08849	3.3349	80	background: ω0 = 0.07584, ω1 = 1.00000, ω2a = 0.07584, ω2b = 1.00000; foreground: ω0 = 0.07584, ω1 = 1.00000, ω2a = 6.21808, ω2b = 6.21808	p0 = 0.63821, p1 = 0.35912, p2a = 0.00171, p2b = 0.00096	-15582.82647	0.632472	0.4264500154	n/a
branch-site model	bats	null	3.08122	3.33427	79	background: ω0 = 0.07232, ω1 = 1.00000, ω2a = 0.07232, ω2b = 1.00000; foreground: ω0 = 0.07232, ω1 = 1.00000, ω2a = 1.00000, ω2b = 1.00000	p0 = 0.58383, p1 = 0.32072, p2a = 0.06161, p2b = 0.03384	-15579.5976			
branch-site model	bats	alternative	3.12988	3.35579	80	background: ω0 = 0.07530, ω1 = 1.00000, ω2a = 0.07530, ω2b = 1.00000; foreground: ω0 = 0.07530, ω1 = 1.00000, ω2a = 10.53445, ω2b = 10.53445	p0 = 0.62612, p1 = 0.33778, p2a = 0.02345, p2b = 0.01265	-15558.2729	42.649394	0	7L (0.917), **24Q (0.998**)**, 27T (0.718), **31E (0.959*)**, 34H (0.887), **35E (0.974*)**, 42E (0.652), 91L (0.888), **298V (0.959*)**, 478W (0.505), 483E (0.768), 549E (0.807), 565P (0.939), **568L (0.998**)**, 569A (0.764), **575G (0.965*)**, 658V (0.810), 771K (0.570)
branch-site model	catarrhines	null	3.08696	3.33682	79	background: ω0 = 0.07190, ω1 = 1.00000, ω2a = 0.07190, ω2b = 1.00000; foreground: ω0 = 0.07190, ω1 = 1.00000, ω2a = 1.00000, ω2b = 1.00000	p0 = 0.59837, p1 = 0.33784, p2a = 0.04077, p2b = 0.02302	-15581.0318			
branch-site model	catarrhines	alternative	3.10444	3.33652	80	background: ω0 = 0.07498, ω1 = 1.00000, ω2a = 0.07498, ω2b = 1.00000; foreground: ω0 = 0.07498, ω1 = 1.00000, ω2a = 8.98776, ω2b = 8.98776	p0 = 0.63077, p1 = 0.35602, p2a = 0.00845, p2b = 0.00477	-15573.75876	14.546074	0.000136773355	206D (0.786), **249M (0.962*)**, 338D (0.581), **653A (0.958*)**, 657K (0.511), **658V (0.957*)**, 706M (0.827), 729P (0.798), 732G (0.616), 788K (0.581)
branch-site model	strepsirrhines	null	3.0744	3.34127	79	background: ω0 = 0.06994, ω1 = 1.00000, ω2a = 0.06994, ω2b = 1.00000; foreground: ω0 = 0.06994, ω1 = 1.00000, ω2a = 1.00000, ω2b = 1.00000	p0 = 0.59047, p1 = 0.30920, p2a = 0.06585, p2b = 0.03448	-15576.45584			
branch-site model	strepsirrhines	alternative	3.08067	3.34233	80	background: ω0 = 0.07170, ω1 = 1.00000, ω2a = 0.07170, ω2b = 1.00000; foreground: ω0 = 0.07170, ω1 = 1.00000, ω2a = 1.38404, ω2b = 1.38404	p0 = 0.60671, p1 = 0.31603, p2a = 0.05080, p2b = 0.02646	-15576.03928	0.83312	0.3613718973	n/a
cladeC model	n/a	M2a_rel (cla	2.99711	3.34639	80	ω0 = 0.03668, ω1 = 1.00000, ω2 = 0.36059	p0 = 0.50433, p1 = 0.27145, p2 = 0.22423	-15575.33101			
cladeC model	n/a	cladeC	3.07137	3.34517	83	ω0 = 0.05915, ω1 = 1.00000, ω2 = 0.08051, ω3 = 1.12260, ω4 = 0.23634, ω5 = 1.34632	p0 = 0.58058, p1 = 0.33089, p2-5 = 0.08853	-15561.96782	26.72638	0.000006718510	n/a

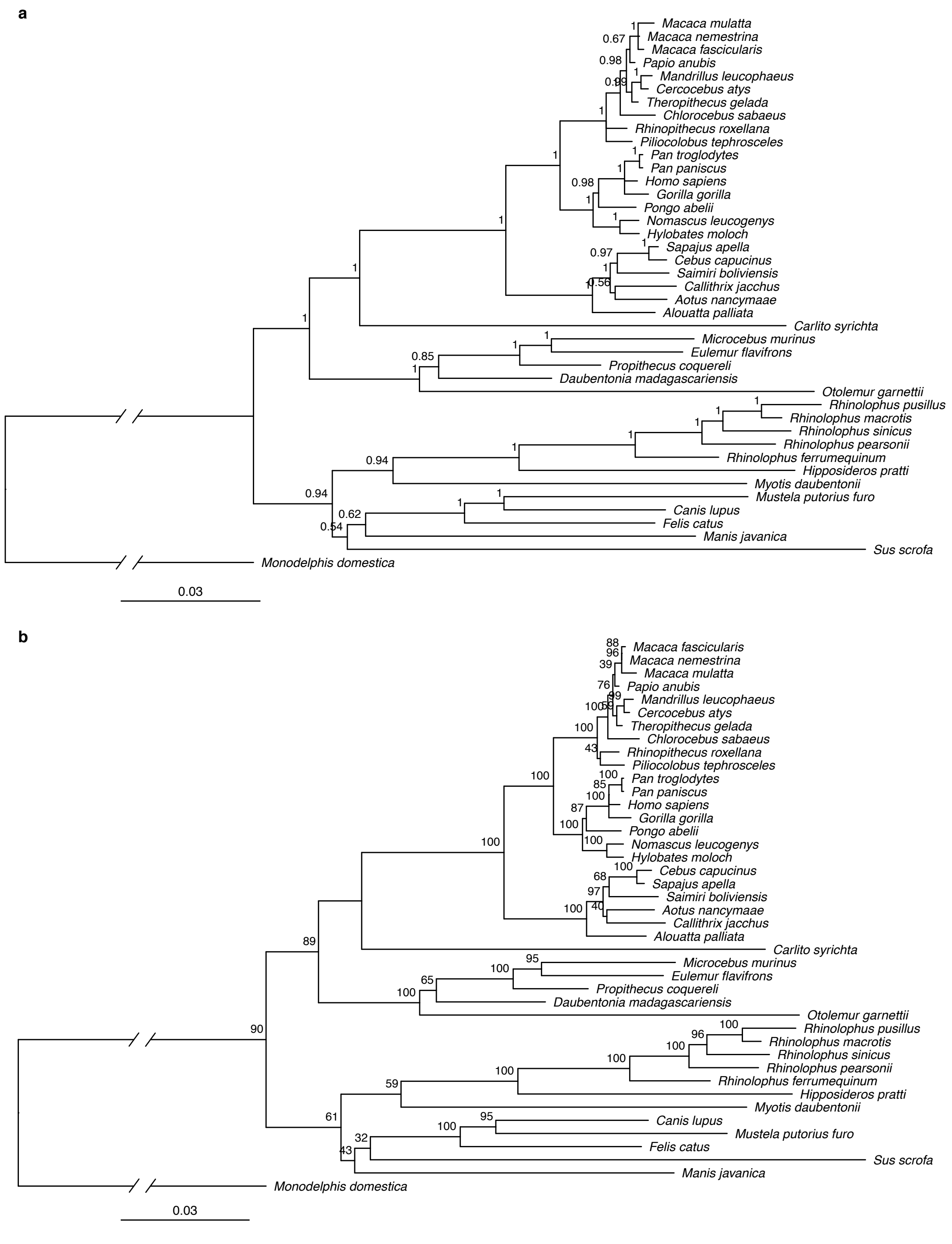

Supplementary Figure S1. *ACE2* gene trees. Trees were built with (a) a Bayesian approach (Mr Bayes[3]) and (b) a maximum likelihood approach (RAxML[4]), using *Monodelphis domestica* as an outgroup. Node labels indicate (a) posterior probabilities or (b) bootstrap support. Scale bars indicate substitutions per site.

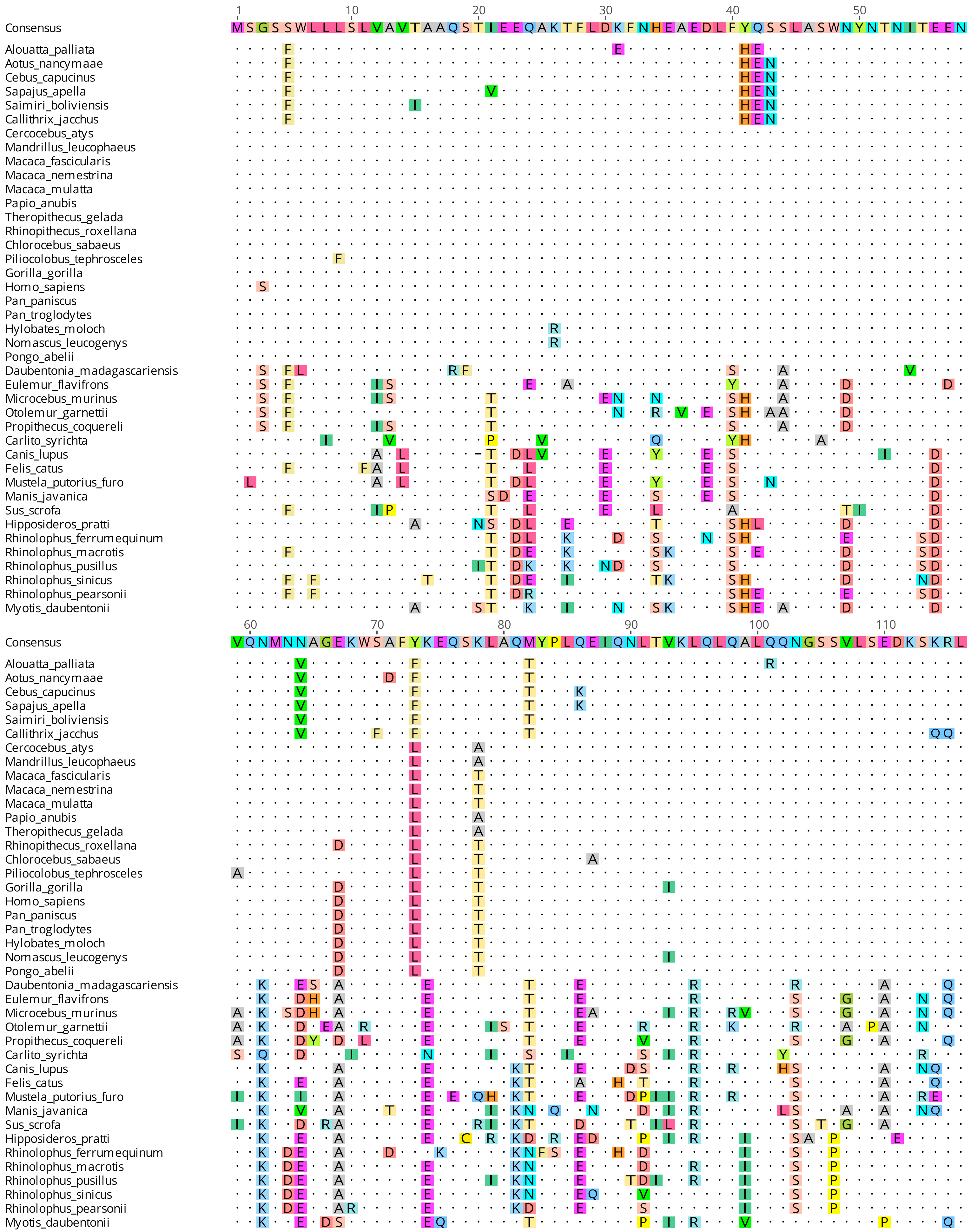

Supplementary Figure S2. Full-length alignment of ACE2 protein sequences.

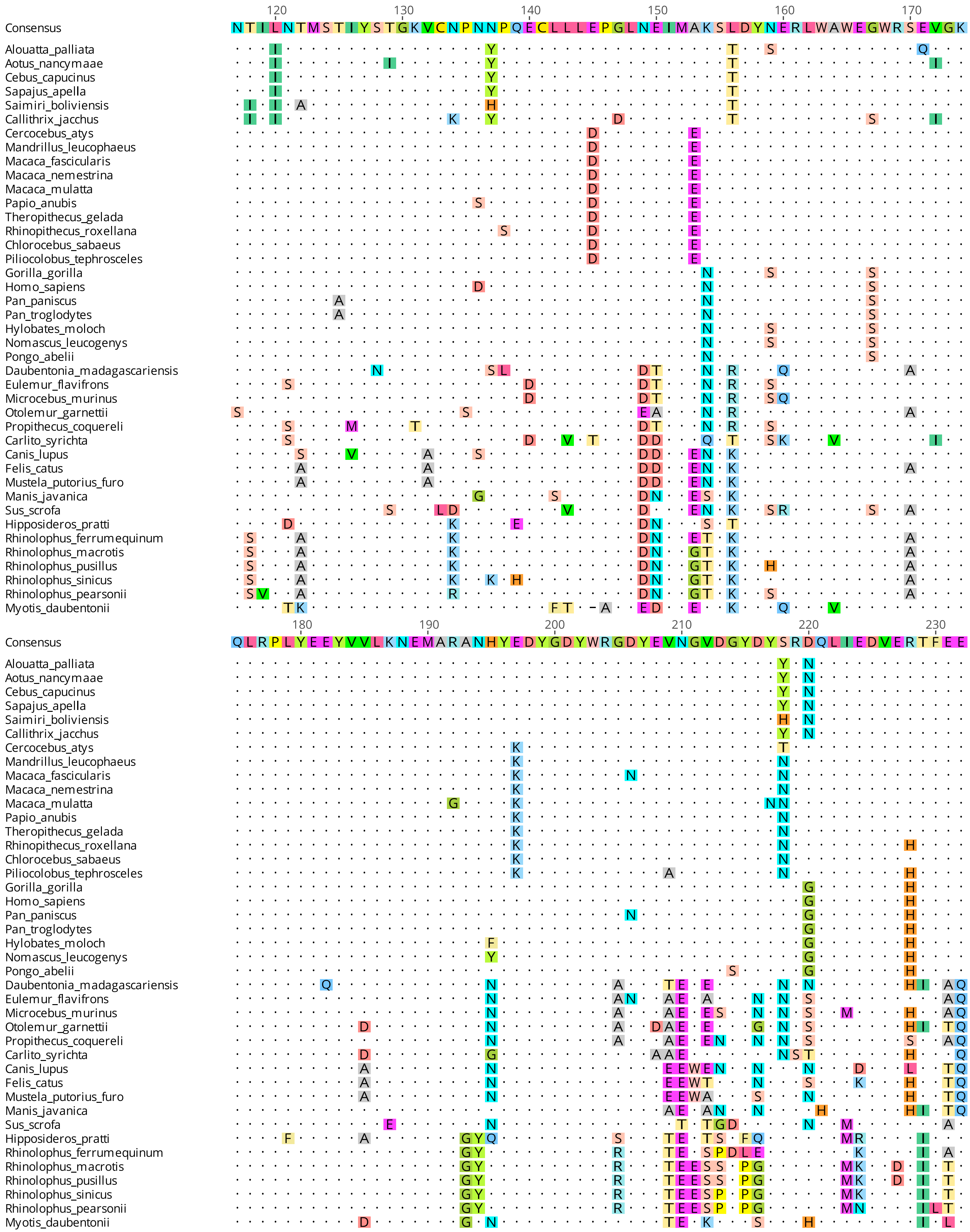

Supplementary Figure S2 cont. Full-length alignment of ACE2 protein sequences.

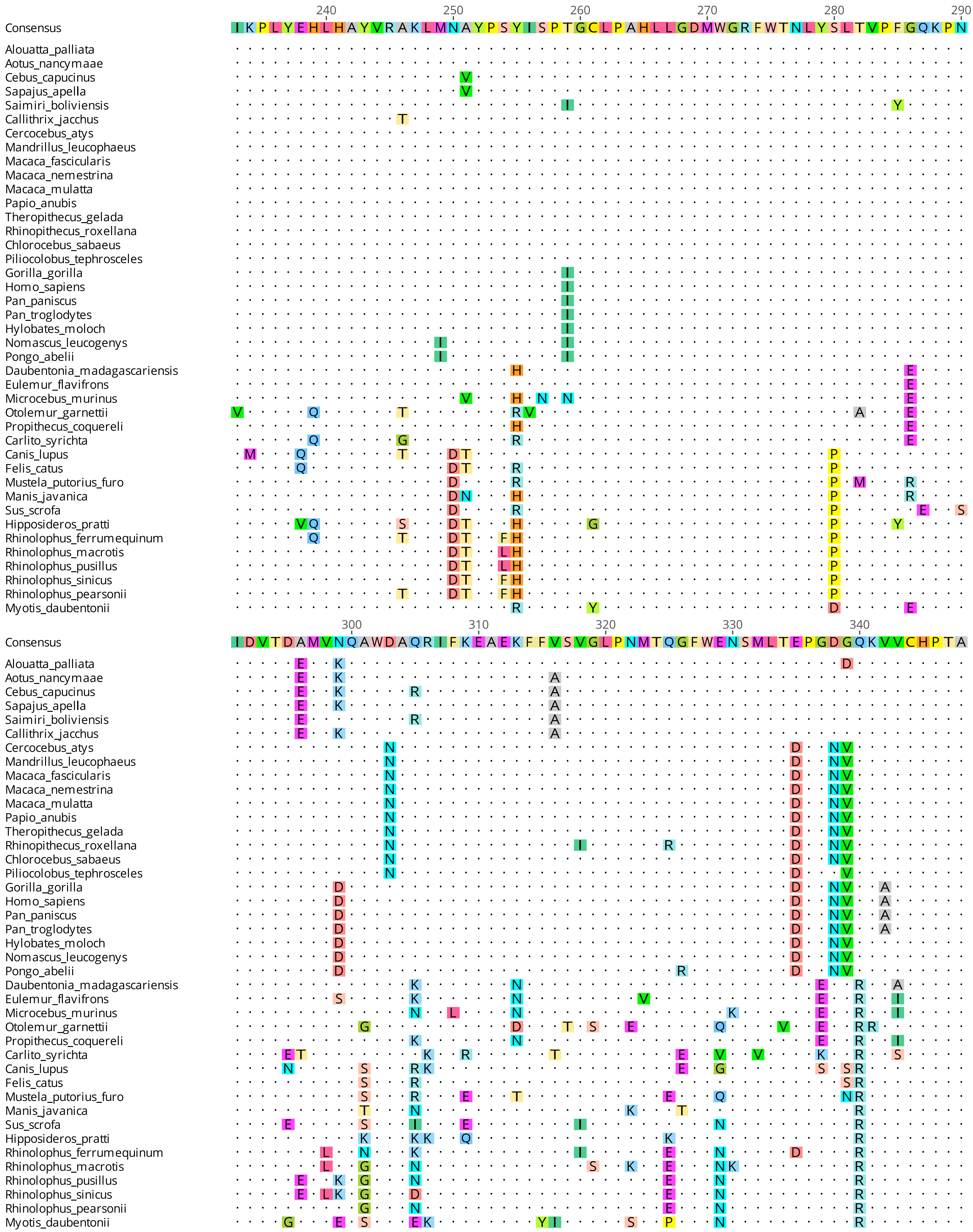

Supplementary Figure S2 cont. Full-length alignment of ACE2 protein sequences.

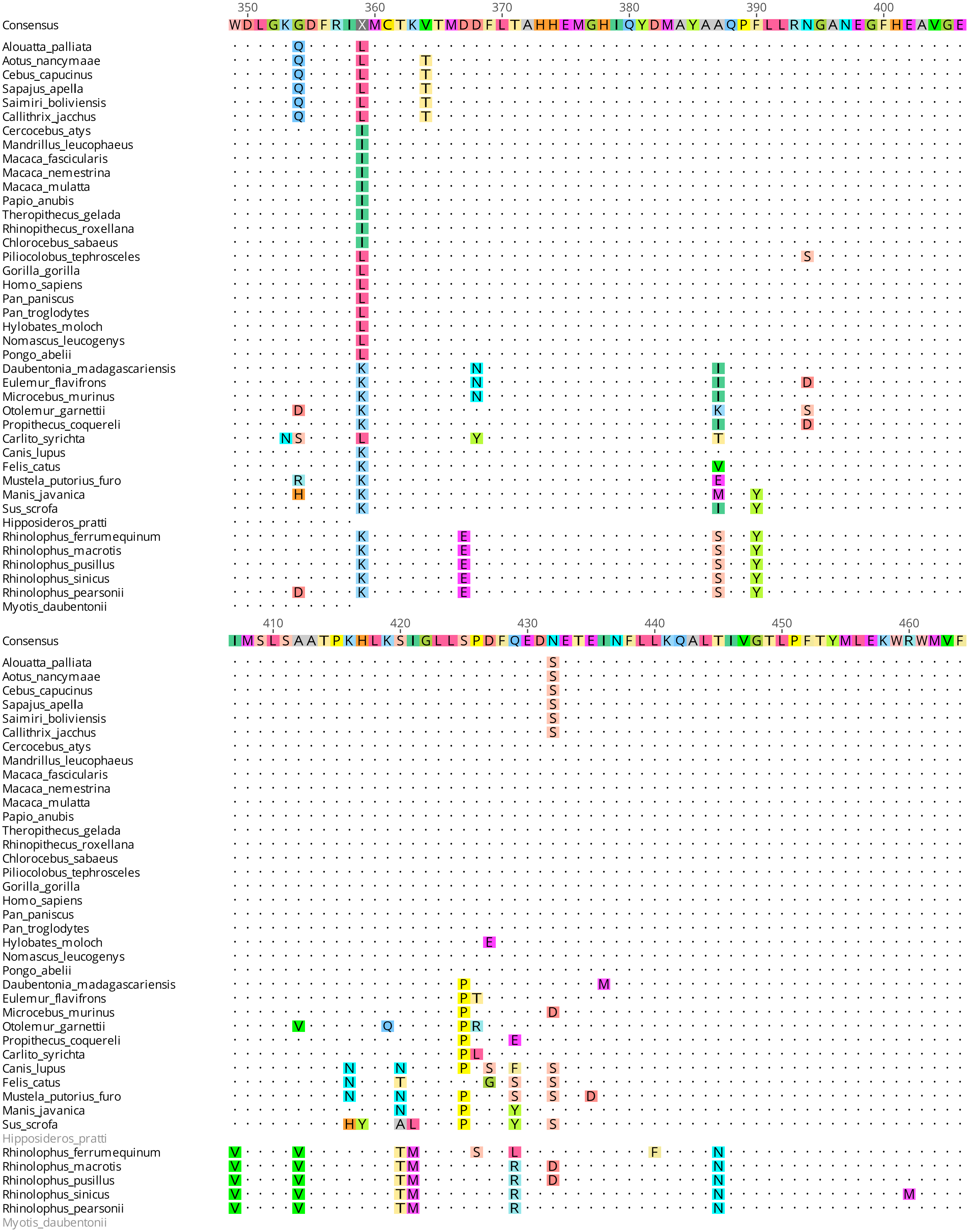

Supplementary Figure S2 cont. Full-length alignment of ACE2 protein sequences.

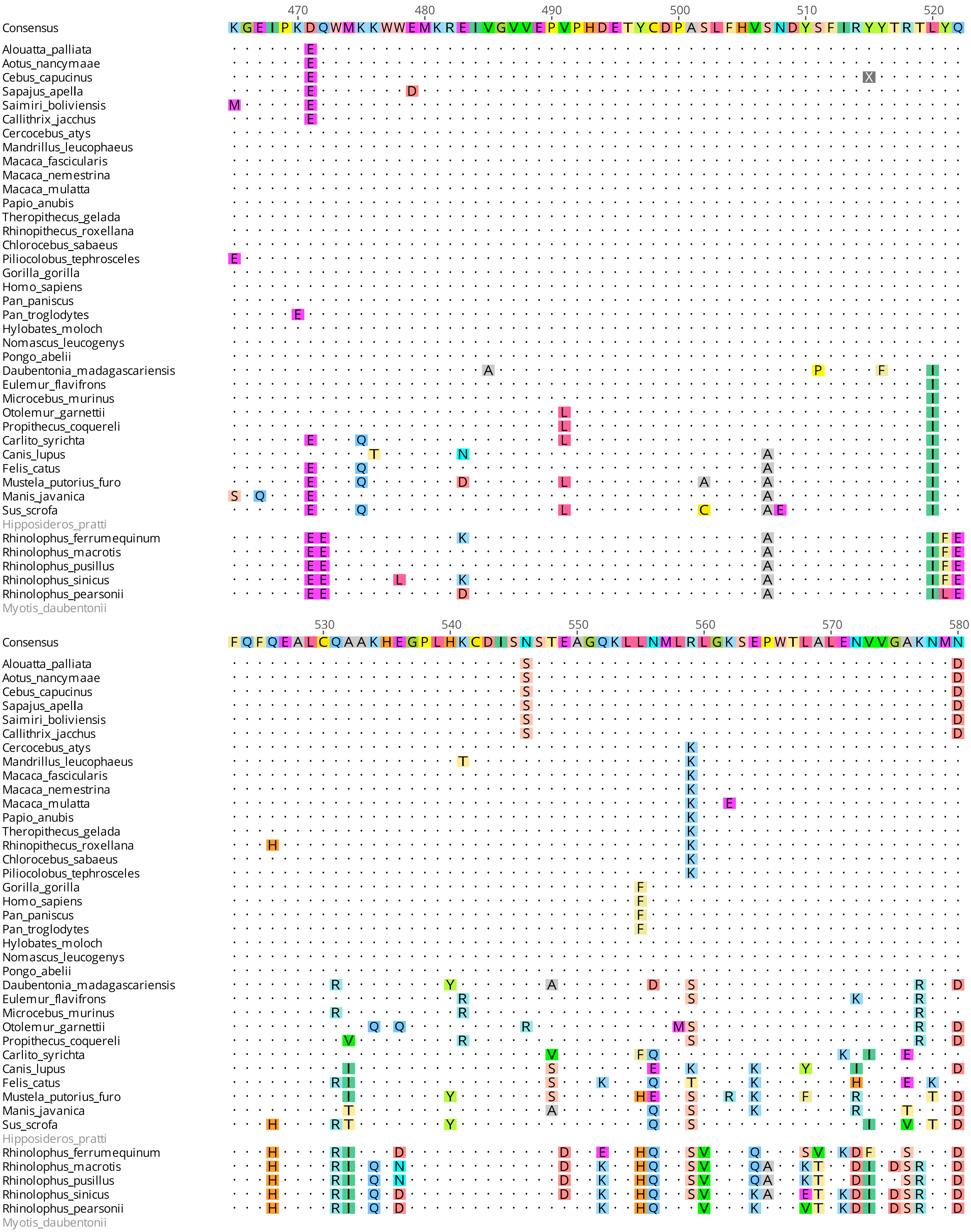

Supplementary Figure S2 cont. Full-length alignment of ACE2 protein sequences.

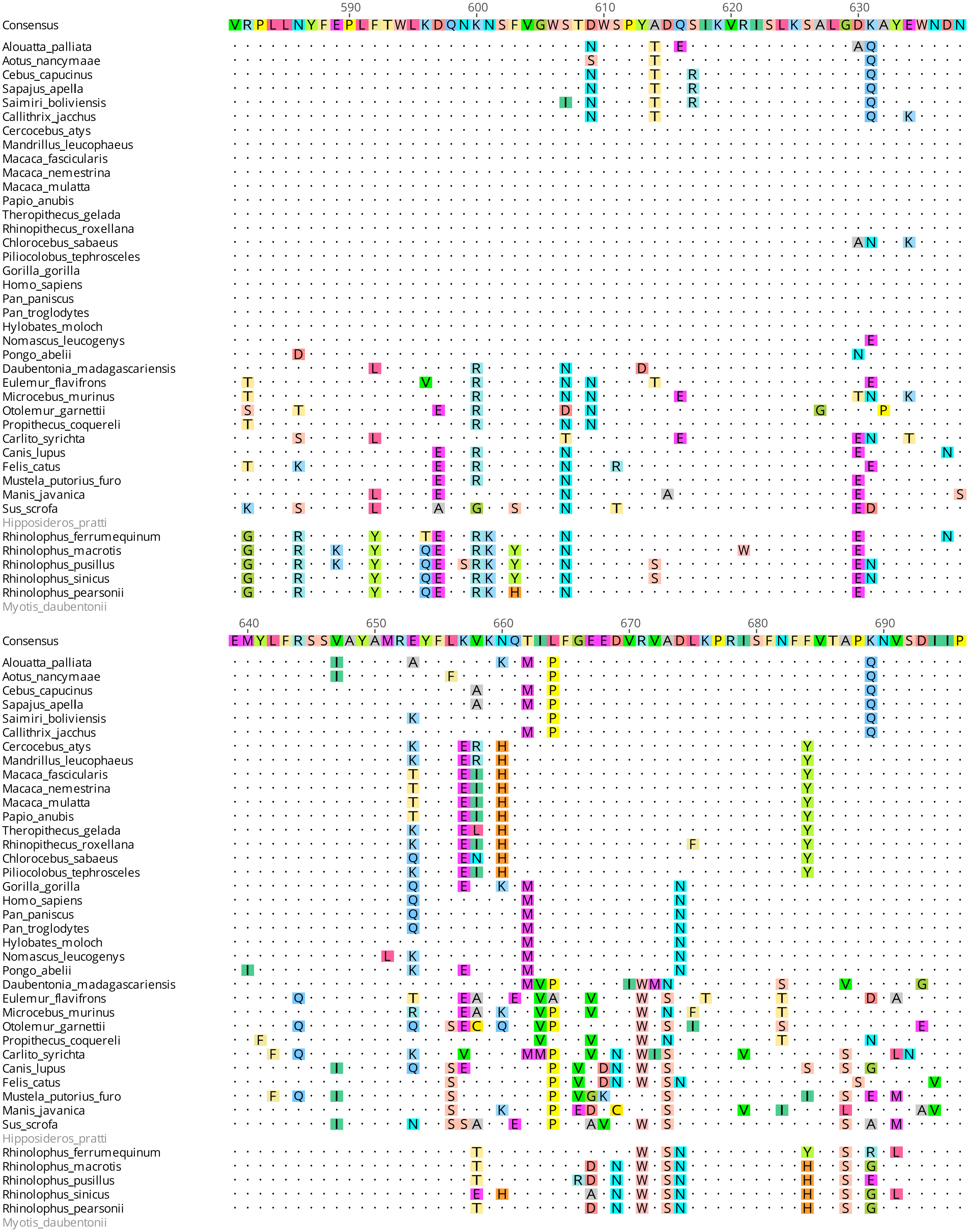

Supplementary Figure S2 cont. Full-length alignment of ACE2 protein sequences.

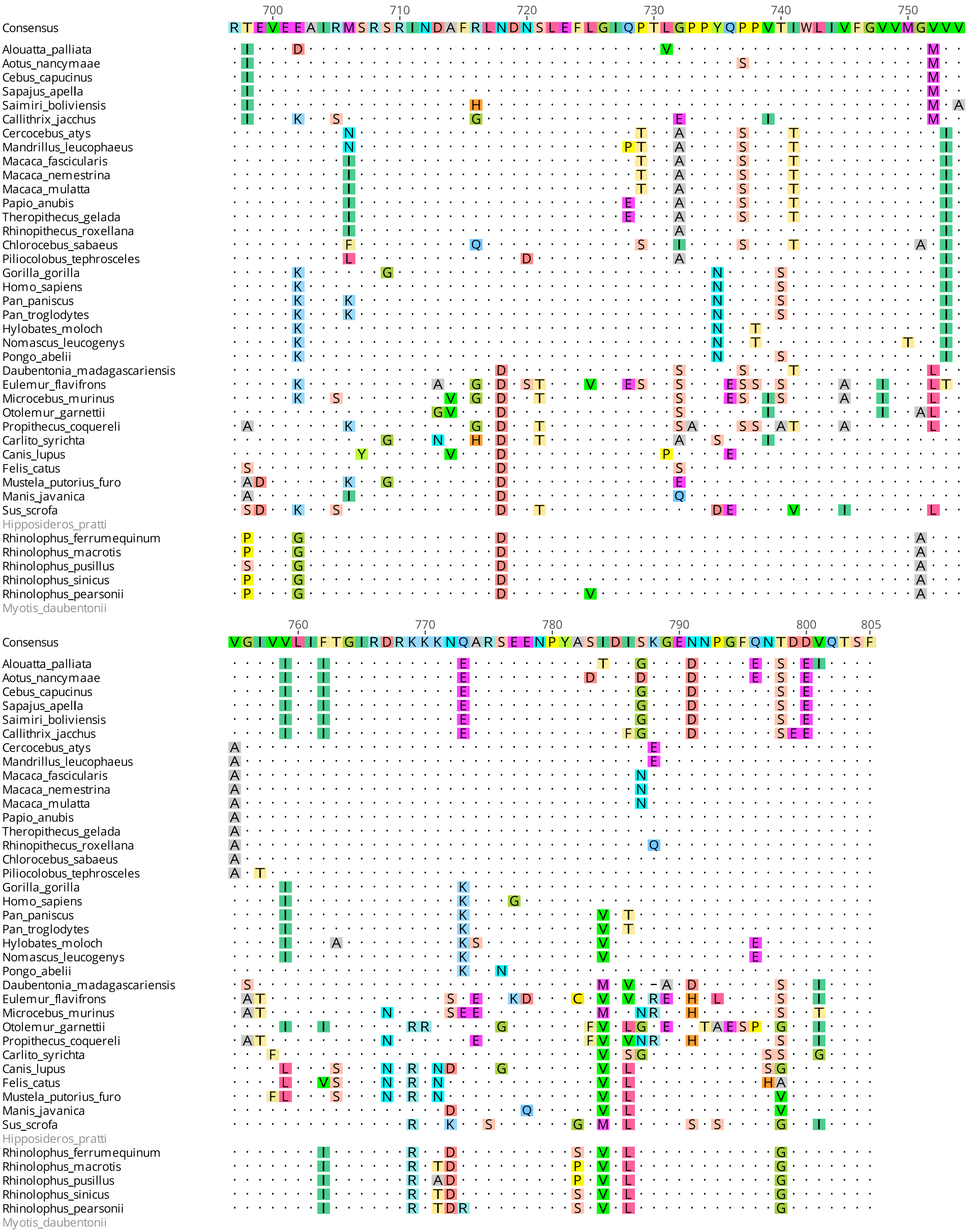

Supplementary Figure S2 cont. Full-length alignment of ACE2 protein sequences.

References

1. Yan, R. *et al.* Structural basis for the recognition of SARS-CoV-2 by full-length human ACE2. *Science* **367**, 1444–1448 (2020).
2. Hou, Y. *et al.* Angiotensin-converting enzyme 2 (ACE2) proteins of different bat species confer variable susceptibility to SARS-CoV entry. *Arch. Virol.* **155**, 1563–1569 (2010).
3. Huelsenbeck, J. P. & Ronquist, F. MRBAYES: Bayesian inference of phylogenetic trees. *Bioinformatics* **17**, 754–755 (2001).
4. Stamatakis, A. RAxML version 8: a tool for phylogenetic analysis and post-analysis of large phylogenies. *Bioinformatics* **30**, 1312–1313 (2014).
