# Supplementary material for: Comparative ACE2 variation and primate COVID-19 risk
Source: Commun Biol. 2020 Oct 27;3:641. doi: 10.1038/s42003-020-01370-w (PMC7591510; doi:10.1038/s42003-020-01370-w)
Supplement: Supplementary file 2 — Description of Additional Supplementary Files [file 42003_2020_1370_MOESM2_ESM.pdf]

Description of Additional Supplementary Files

File Name: Supplementary Data 1
Description: Pairwise identities of ACE2 nucleotide sequences between all species in our study. Number indicates the percentage of bases that are identical across the full length of the gene sequence.

File Name: Supplementary Data 2
Description: Pairwise identities of ACE2 protein sequences between all species in our study. Number indicates the percentage of residues that are identical across the full length of the protein sequence.

File Name: Supplementary Data 3
Description: Pairwise similarities of ACE2 protein sequences between all species in our study. Number indicates the percentage of residues that are similar (score≥1 in the BLOSUM62 matrix) across the full length of the protein sequence.

File Name: Supplementary Data 4
Description: ACE2 nucleotide sequences in fasta format.

File Name: Supplementary Data 5
Description: ACE2 protein sequences in fasta format.

File Name: Supplementary Data 6
Description: Alignment of ACE2 nucleotide sequences in fasta format.

File Name: Supplementary Data 7
Description: Alignment of ACE2 protein sequences in fasta format.
