# Supplementary material for: Comparative ACE2 variation and primate COVID-19 risk
Source: Commun Biol. 2020 Oct 27;3:641. doi: 10.1038/s42003-020-01370-w (PMC7591510; doi:10.1038/s42003-020-01370-w)
Supplement: Supplementary file 10 — Reporting summary [file 42003_2020_1370_MOESM10_ESM.pdf]

nature research

Corresponding author(s): Amanda Melin; James Higham

Last updated by author(s): Sep 10, 2020

Reporting Summary

Nature Research wishes to improve the reproducibility of the work that we publish. This form provides structure for consistency and transparency in reporting. For further information on Nature Research policies, see Authors & Referees and the Editorial Policy Checklist .

Statistics

For all statistical analyses, confirm that the following items are present in the figure legend, table legend, main text, or Methods section.

n/a	Confirmed	
☐	☒	The exact sample size (*n*) for each experimental group/condition, given as a discrete number and unit of measurement
☒	☐	A statement on whether measurements were taken from distinct samples or whether the same sample was measured repeatedly
☐	☒	The statistical test(s) used AND whether they are one- or two-sided<br>*Only common tests should be described solely by name; describe more complex techniques in the Methods section.*
☒	☐	A description of all covariates tested
☒	☐	A description of any assumptions or corrections, such as tests of normality and adjustment for multiple comparisons
☒	☐	A full description of the statistical parameters including central tendency (e.g. means) or other basic estimates (e.g. regression coefficient) AND variation (e.g. standard deviation) or associated estimates of uncertainty (e.g. confidence intervals)
☐	☒	For null hypothesis testing, the test statistic (e.g. *F*, *t*, *r*) with confidence intervals, effect sizes, degrees of freedom and *P* value noted<br>*Give P values as exact values whenever suitable.*
☐	☒	For Bayesian analysis, information on the choice of priors and Markov chain Monte Carlo settings
☒	☐	For hierarchical and complex designs, identification of the appropriate level for tests and full reporting of outcomes
☒	☐	Estimates of effect sizes (e.g. Cohen's *d*, Pearson's *r*), indicating how they were calculated

Our web collection on statistics for biologists contains articles on many of the points above.

Software and code

Policy information about availability of computer code

Data collection	Entrez Direct (EDirect Version 10.1) e-utilities from NCBI were used to retrieve genetic sequences from NCBI's GenBank after manually collecting the relevant accession numbers. Code used for sequence retrieval is available in the project's GitHub repository (https://github.com/MareikeJaniak/ACE2); NCBI Blast 2.10.0 was used to identify ACE2 in an unpublished Alouatta palliata genome.<br><br>PDB file 6M0J was analyzed by SSIPe server (https://zhanglab.ccmb.med.umich.edu/SSIPe/) and PyRosetta (http://www.pyrosetta.org).
Data analysis	Coding sequences were translated using Geneious Version 9.1.8 and we aligned the amino acid sequences with MAFFT. We reconstructed trees using both Bayesian (MrBayes 3.2.6) and Maximum Likelihood (RAxML 8.2.11) methods with 200,000 MCMC cycles and 1,000 bootstrap replicates, respectively (code available on GitHub: https://github.com/MareikeJaniak/ACE2 (2020)). Gene trees were compared to a current species phylogeny assembled using TimeTree (timetree.org accessed April 3). The computational alanine mutagenesis analysis was performed with Rosetta Software and PDB file 6M0J. Impacts of mutations at critical binding sites were analyzed with SSIPe server (https://zhanglab.ccmb.med.umich.edu/SSIPe/) and PDB file 6M0J. CodeML models implemented in PAML 4.8 were used to test for evidence of positive selection acting on ACE2.

For manuscripts utilizing custom algorithms or software that are central to the research but not yet described in published literature, software must be made available to editors/reviewers. We strongly encourage code deposition in a community repository (e.g. GitHub). See the Nature Research guidelines for submitting code & software for further information.

Data

Policy information about availability of data

All manuscripts must include a data availability statement. This statement should provide the following information, where applicable:

- Accession codes, unique identifiers, or web links for publicly available datasets
- A list of figures that have associated raw data
- A description of any restrictions on data availability

Nucleotide and protein sequences used in this study are available from NCBI and are also available as fasta files and alignments on Github (https://github.com/MareikeJaniak/ACE2). The data availability statement describes this and points to Supplementary Table S2 for accession numbers. The genome of Alouatta palliata from which this species' ACE2 sequence was retrieved is unpublished, but it's ACE2 sequence is now available within the fasta files and alignments in the GitHub repository. The full genome can be made available upon request to MCJ. All code used in this project is available in the aforementioned GitHub repository.

Field-specific reporting

Please select the one below that is the best fit for your research. If you are not sure, read the appropriate sections before making your selection.

☐ Life sciences ☐ Behavioural & social sciences ☒ Ecological, evolutionary & environmental sciences

For a reference copy of the document with all sections, see nature.com/documents/nr-reporting-summary-flat.pdf

Ecological, evolutionary & environmental sciences study design

All studies must disclose on these points even when the disclosure is negative.

Study description	This study has two main components. The first involves comparative genetics. We compiled ACE2 gene sequence data from 29 primate species, one sequence per species, along with ACE2 sequences other mammals relevant to COVID-19 emergence and susceptibility. We assessed the amino acid residues identified as critical for ACE2 recognition by the SARS-CoV-2 virus.<br><br>Secondly, we undertook protein modeling to gauge the likely effect of the amino acid differences identified in step 1. We used computational alanine scanning mutagenesis to probe the contribution of each interfacial residue to binding and we used an algorithm to assess of residue differences seen among species.
Research sample	We sampled all available primate genomes that were sequenced in high coverage and well annotated. We cover the breadth of primate taxa in this sample. For comparison, we assessed 4 species of other mammals that have been tested directly for SARS-CoV-2 susceptibility in laboratory infection studies (ferret, dog, cat, pig). We also included in our analysis the amino acid sequence variation at these sites for horseshoe bats, thought to be the original vector of the virus, and pangolins, a potential intermediate host, where viral recombination may have led to the novel viral form SARS-CoV-2. Data on the age and sex of the samples were not considered and we consider these sequences to representative of the species.
Sampling strategy	We included all primates with publicly available reference genomes to capture to the best of our ability the diversity of the Order. The species covered represent well total primate diversity, and are sufficient for assessing high risk to catarrhines, our principle finding.
Data collection	ACE2 gene sequences were downloaded, aligned and manually inspected by MCJ, who then corrected any misalignments, and verified the absence of indels and premature stop codons.<br><br>Individual residues in PDB file 6M0J were mutated in UCSF Chimera using the Dynameomics rotamer library. Files were manually inspected and analyzed with PyRosetta for alanine scanning analysis.
Timing and spatial scale	Genomic data were downloaded on April 2nd and April 3rd. The protein models with reconstructed sequence were run April 7-9th.
Data exclusions	We excluded Hipposideros pratti and Myotis daubentonii from PAML analyses, because only a partial ACE2 sequence was available for these two species.
Reproducibility	For generation of gene trees we ran 1,000 bootstrap replicates (RAxML) and 200,000 MCMC cycles (MrBayes) to generate robust relationships with confidence metrics reported.
Randomization	Not applicable
Blinding	Not applicable

Did the study involve field work? ☐ Yes ☒ No

Reporting for specific materials, systems and methods

We require information from authors about some types of materials, experimental systems and methods used in many studies. Here, indicate whether each material, system or method listed is relevant to your study. If you are not sure if a list item applies to your research, read the appropriate section before selecting a response.

Materials & experimental systems

n/a	Involved in the study
☒	☐ Antibodies
☒	☐ Eukaryotic cell lines
☒	☐ Palaeontology
☐	☒ Animals and other organisms
☒	☐ Human research participants
☒	☐ Clinical data

Methods

n/a	Involved in the study
☒	☐ ChIP-seq
☒	☐ Flow cytometry
☒	☐ MRI-based neuroimaging

Animals and other organisms

Policy information about studies involving animals; ARRIVE guidelines recommended for reporting animal research

Laboratory animals	n/a
Wild animals	n/a
Field-collected samples	n/a
Ethics oversight	No ethics approval was necessary as we conducted analysis of publicly available genomes and did not collect new data.

Note that full information on the approval of the study protocol must also be provided in the manuscript.
